# Adipose-Derived Stem Cell Therapy Combined With Platelet-Rich Plasma for the Treatment of Avascular Necrosis of the Talus

**DOI:** 10.7759/cureus.77578

**Published:** 2025-01-17

**Authors:** Rui Sousa, Jaime Babulal, Paulo Amado

**Affiliations:** 1 Orthopaedics and Traumatology Department, Unidade Local de Saúde de Viseu Dão-Lafões, Viseu, PRT; 2 Orthopaedics and Traumatology Department, Unidade Local de Saúde do Médio Ave, Vila Nova de Famalicão, PRT; 3 Orthopaedics Department, Lusíadas Porto and Lusíadas Santa Maria da Feira Hospitals, Porto, PRT

**Keywords:** avascular necrosis, case report, platelet-rich plasma (prp), stem cell therapy, stromal vascular fraction (svf)

## Abstract

Avascular necrosis (AVN) is characterized by compromised blood supply to bone tissue, often resulting in significant pain and functional impairment. Conservative treatment options for early-stage AVN of the talus are scarce, with non-weight-bearing protocols and hyperbaric oxygen therapy showing limited efficacy. This report presents a potential novel approach to managing non-traumatic AVN of the talus using a combination of adipose-derived stromal vascular fraction (SVF) and platelet-rich plasma (PRP). A 60-year-old male presented with progressive right ankle pain of a two-year evolution. Magnetic resonance imaging (MRI) revealed stage II AVN of the talus with extensive bone infarctions occupying almost all of its volume but with no collapse. A single injection of SVF and PRP was performed after diagnostic arthroscopy, followed by a tailored rehabilitation protocol. The patient experienced significant pain relief, with no further need for analgesics, and was able to return to non-impact sports. At the 18-month follow-up, functional scores showed significant improvement, and MRI showed stable AVN with no evidence of collapse. This report highlights SVF and PRP as a potential effective conservative option for non-traumatic talar AVN.

## Introduction

Talar avascular necrosis (AVN) presents a significant clinical challenge, as even with meticulous management, outcomes and overall patient well-being can vary widely. Similar to other forms of AVN, this condition is fundamentally characterized by compromised blood supply to the talar bone, leading to progressive bone necrosis and joint collapse. In recent years, numerous surgical techniques for managing talar AVN have been introduced. However, effective conservative treatment options are limited, particularly in non-traumatic cases, with non-weight-bearing and hyperbaric oxygen therapy still being the most effective approaches for early-stage disease [1]. As a result of this scarcity of conservative treatment options, attention has been driven to other novel and potential approaches.

Orthobiologic therapies, such as the combination of adipose tissue-derived stem cells (ASDCs) and platelet-rich plasma (PRP), have gained widespread use over the past decade. This combination aims to harness the regenerative and immunomodulatory potential of stem cells, along with the anti-inflammatory properties of PRP, to enhance healing and modulate disease progression across a range of orthopedic conditions.

Promising results have been demonstrated in the treatment of hip AVN with bone marrow-derived mesenchymal stem cells (MSCs) or ADSCs combined with PRP, with reports indicating the presence of sustained, regenerated medullary bone-like tissue in severely necrotic femoral heads following the injection of this combination [2,3]. Similarly, in knee osteoarthritis (OA), orthobiologics have been developed to offer more than just symptom reduction, showing promising results in slowing down disease progression in animal OA models. Among these, PRP is demonstrated to offer better results than other traditional products [4].

In this article, we report the case of a patient with non-traumatic AVN of the talus who underwent treatment with stromal vascular fraction (SVF) combined with PRP, with excellent clinical results. While no clinical trials have been conducted for talar AVN, literature regarding its effectiveness in other conditions provides a strong rationale for exploring this combination as a conservative treatment option in this case.

## Case presentation

A 60-year-old male, with a medical history of hypertension and dyslipidemia, managed with a beta-blocker and a statin, presented with persistent and progressive right ankle pain with an approximately two-year evolution. On physical examination, a mild restriction in dorsiflexion and mechanical anterior joint pain was noted, mainly during weight-bearing. Before the onset, the patient was actively engaged in multiple sports such as mountain running (approximately 10 km runs for several days a week), diving, and cycling. The clinical onset was during the COVID-19 pandemic lockdown when the patient stopped exercising and noticed progressive pain when he restarted engaging in night strolls. The worsening of the pain ultimately led to an inability to practice sports. He reported two COVID-19 infections during this period.

**Figure 1:**
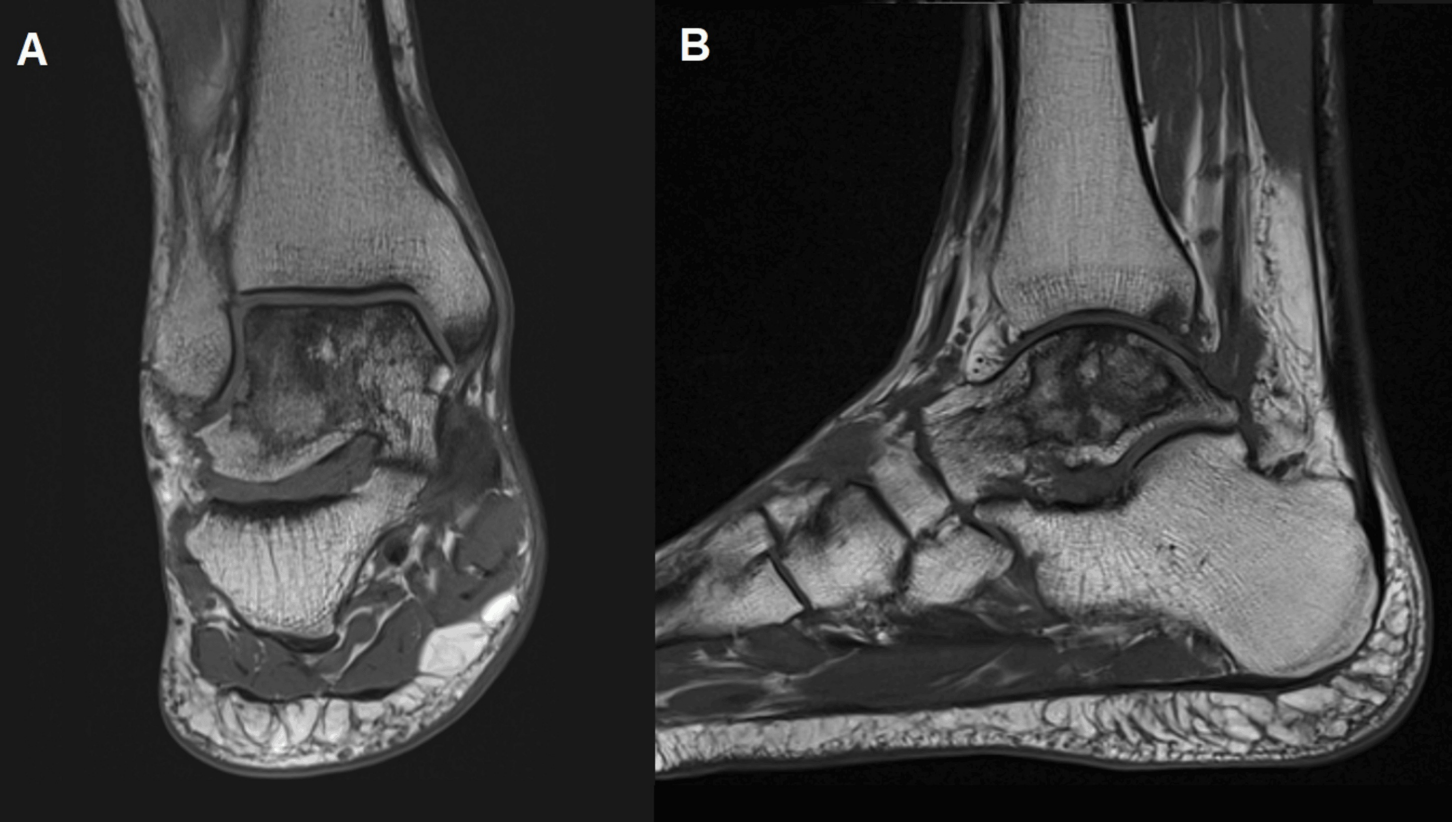
MRI T1-weighted images of the ankle in coronal (A) and sagittal view (B) at the time of the first evaluation. MRI exhibiting extensive bone infarctions of the talar head and body, occupying almost all of its volume. Focal chondropathy of the talus and distal tibial could also be identified.

Magnetic resonance imaging (MRI) revealed stage II AVN of the talus with extensive bone infarctions in the head and body, occupying almost all of its volume. The necrotic areas showed replacement by fibrosis and fat, without collapse, fractures, or cavitated lesions. Focal chondropathy in the talus and distal tibial could also be identified (Figure 1). The patient was a non-smoker and had a body mass index of 21.7 kg/m², classifying him as within the normal weight range.

Before our evaluation, the patient had previously undergone hyperbaric oxygen therapy in three six-month periods at another institution, resulting in minimal symptomatic relief. During this period, he had also been taking daily oral analgesics, including non-steroidal anti-inflammatory drugs (NSAIDs) and opioids. At presentation, pain management involved morphine patches, which were the only remaining option enabling him to perform his basic activities of daily living. Functional assessments included an American Orthopaedic Foot & Ankle Society (AOFAS) Ankle-Hindfoot Scale score of 36, a Visual Analog Scale (VAS) score of 10, and Foot and Ankle Ability Measure (FAAM) scores of 26/84 (31%) for the Activities of Daily Living Subscale and 0/32 (0%) for the Sports Subscale.

**Figure 2:**
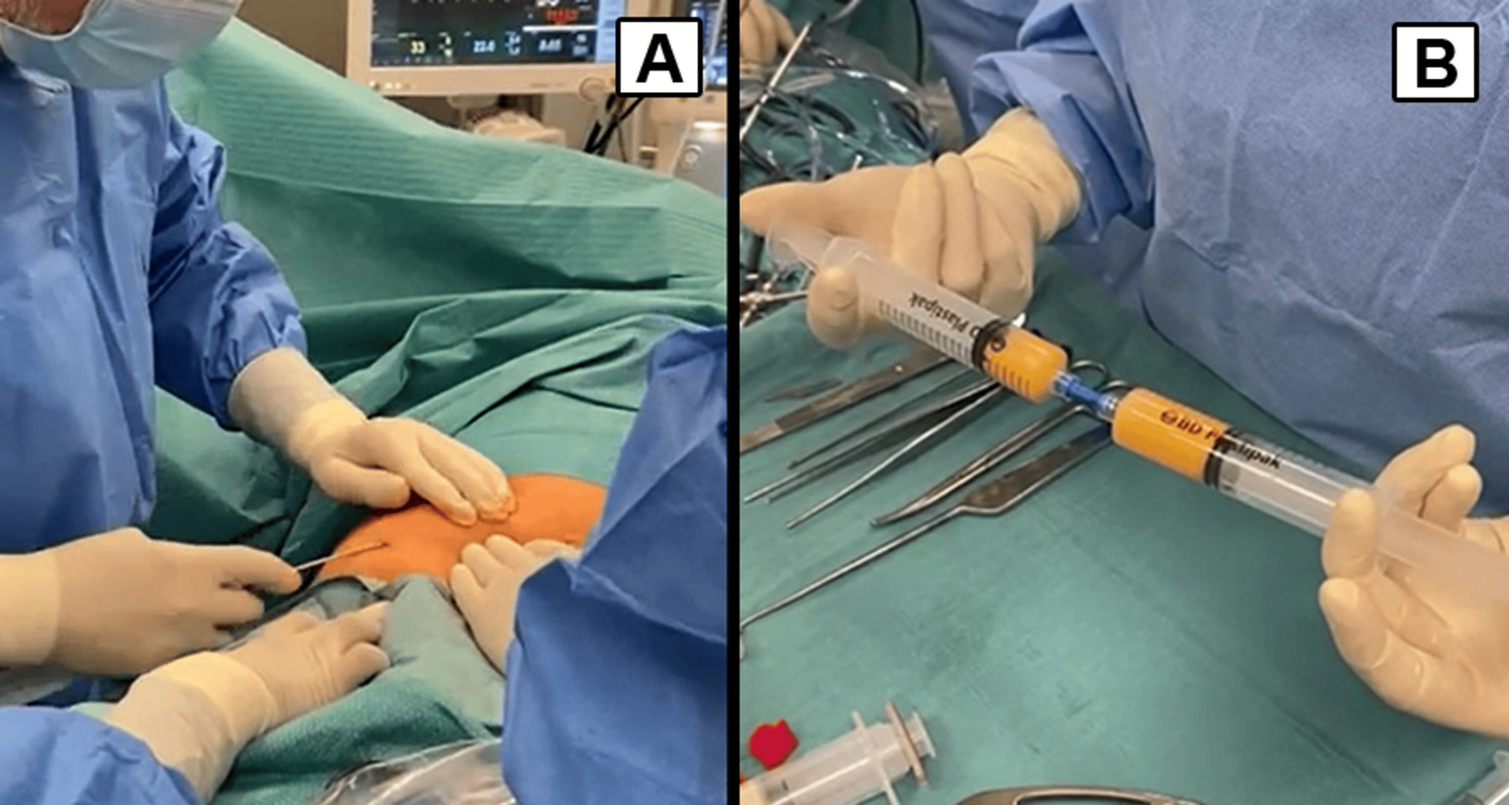
Isolation and preparation of adipose-derived stromal vascular fraction (SVF). A. Harvesting of adipose tissue performed via subcutaneous liposuction from the lower abdominal area. B. Microfat processing for the isolation of SVF. Mechanical dissociation of adipocytes during microfat processing facilitates the extraction and concentration of the regenerative cells within the SVF, allowing its collection as a pellet.

After reviewing treatment options, an injection of a combination of SVF and PRP was proposed. The patient was advised not to take NSAIDs or aspirin one week before and after the procedure. In the operating room, approximately 30 mL of adipose tissue was harvested via subcutaneous liposuction from the lower abdominal area under sterile conditions (Figure 2A). After harvesting, the microfat was obtained after a single centrifugation. The SVF was isolated by additional processing of the microfat, with further mechanical dissociation of the adipocytes whereby the regenerative cells within the SVF could be collected as a pellet (Figure 2B). PRP was prepared from autologous blood using a similar centrifugation method, achieving a platelet concentration 2.5 times higher than normal.

A diagnostic arthroscopy was performed to evaluate cartilage status, identifying grade II lesions on the Outerbridge classification. The combination of SVF and PRP was then introduced into the ankle joint in a controlled manner following this diagnostic procedure.

The post-procedure protocol included a restricted weight-bearing period allowing full ankle mobilization for four weeks, followed by progression to full weight-bearing. The patient experienced significant clinical improvement, requiring no pain medication from the early post-procedure period onwards, which profoundly enhanced his quality of life. He showed sustained symptomatic relief, having resumed non-impact sports activity, such as cycling, and remaining clinically stable at 18 months of follow-up.

**Figure 3:**
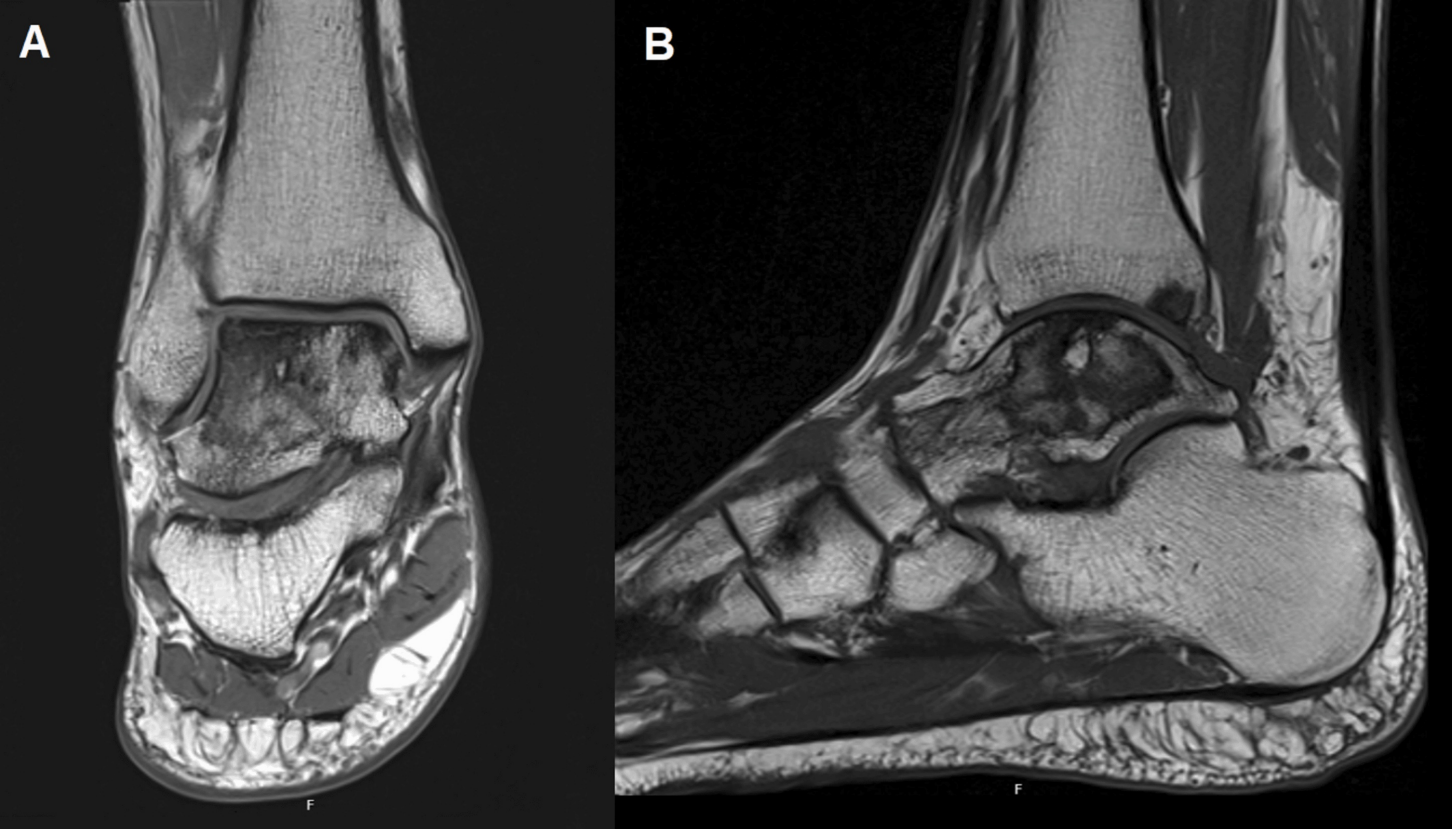
MRI T1-weighted images of the ankle in coronal (A) and sagittal view (B) at the 18-month follow-up. Comparison MRI revealing a relatively stable avascular necrosis with a small insufficiency fracture of the lateral trochlea but no evidence of collapse.

At this follow-up, a comparison MRI revealed a relatively stable AVN with a small insufficiency fracture of the lateral trochlea but no evidence of collapse (Figure 3). Functional scores reflected this clinical improvement, with an AOFAS score of 77, a VAS score of 5, and FAAM scores of 55/84 (65%) for the Activities of Daily Living Subscale and 4/32 (13%) for the Sports Subscale. The patient remains off baseline analgesics, reporting only slight pain and discomfort during daily activities.

## Discussion

Cases of non-traumatic AVN of the talus have been reported in patients treated chronically with corticosteroids; those suffering from alcoholism, systemic lupus erythematosus (SLE), sickle cell anemia, and hyperlipidemia; and those who have undergone renal transplants or irradiation. While most studies on AVN of the talus address cases with a traumatic onset, research on non-traumatic onset remains scarce, particularly regarding conservative treatment options.

The natural history and evolution of this condition are characterized by a continuous lack of oxygen supply to the affected area, causing the necrotic bone to surpass the threshold at which its structural integrity can be maintained and ultimately leading to subchondral bone collapse and joint incongruity. As such, non-weight-bearing has been described as the first line of treatment mostly for patients with post-traumatic talar AVN in stages I and II. The effectiveness of solely non-weight-bearing ranges from 29% to 55%, with the possibility of enhancement to up to 77% using liquid-electric extracorporeal shockwave therapy. Hyperbaric oxygen therapy has also been described as effective in the early postoperative period in a post-traumatic setting. Core decompression is the first-line surgical option, mainly for early-stage non-traumatic talus AVN, followed by bone grafting and joint-sacrificing surgical procedures [1,5-7].

The use of orthobiologic therapies, including SVF and PRP, provides a novel approach to conservative treatment, supported by promising findings in other orthopedic conditions. Several studies have explored the use of SVF and PRP in different joints and conditions, demonstrating their potential benefits. While most studies have focused on hip AVN and knee OA, the promising results observed in these areas provide a basis for considering their application in other challenging orthopedic conditions, such as AVN of the talus.

The combination of adipose-derived mesenchymal stem cells (ADSCs) and PRP has been shown to induce sustained regeneration of medullary bone-like tissue in severely necrotic femoral heads, with one report of complete resolution in early-stage disease [2,3]. Bone marrow aspirate injections have also been described as a viable option for non-weight-bearing augmentation treatment and have been shown to reduce the rate of collapse (progression to stage III) in post-traumatic femoral head AVN [1]. However, no similar clinical trials on talar AVN have been conducted.

Hernigou et al. compared the outcomes of treating post-traumatic osteonecrosis of the talus before collapse with percutaneous injection of autologous bone marrow concentrate from the iliac crest in the talus after minimal core decompression with core decompression alone, concluding that, in the first group, collapse frequency was lower and follow-up showed longer duration of survival before collapse or arthrodesis. The first group also afforded a significantly greater reduction in pain and joint symptoms, regardless of the size and stage of the osteonecrosis. Furthermore, the time to successfully achieve fusion after arthrodesis was significantly shorter compared with the other ankles, which had core decompression alone [8].

Adipose tissue is emerging as the source of choice to obtain MSCs owing to the advantages compared to other sources such as bone marrow. Adipose tissue can be harvested with a low discomfort and allows obtaining a high concentration of MSCs with immunomodulatory and anti-inflammatory properties. The use of adipose tissue-derived injectable products in clinical practice is growing for the management of OA joints, with most of the literature comprising studies in the knee joint, and preliminary clinical studies reporting their safety and efficacy [4,9-11]. Several studies have reported that the combined use of adipose tissue-derived products and PRP provides an overall clinical improvement in pain and function in OA patients, with similar results obtained for different joints evaluated [12-15].

Hurley et al. conducted a systematic review on the use of ADSCs in OA, concluding that all studies reported improvements in clinical outcomes with the use of SVF. However, while SVF appears to yield favorable clinical results with minimal risk of side effects in OA management, the variability in the data and the concurrent use of biological adjuvants have complicated the assessment of ADSCs’ standalone effectiveness [16].

ADSCs have also been described to be effective in cartilage regeneration. Kim et al. compared clinical and second-look arthroscopic outcomes in patients undergoing arthroscopic marrow stimulation combined with lateral sliding calcaneal osteotomy for varus ankle OA, with or without MSCs injection, describing significant improvements in VAS and AOFAS scores, as well as better International Cartilage Repair Society (ICRS) grades, at short-term follow-up after marrow stimulation with additional MSC injection compared with after marrow stimulation alone [17].

Interest in ADSCs has grown significantly in recent years, and they are now being used in clinical settings for a variety of orthopedic applications in human patients. Due to their potential to regenerate cartilage, bone, and tendons, SVFs are being explored as a treatment option for other conditions such as chondromalacia, meniscus tears, and tendon injuries. These applications have demonstrated potential benefits, with no reports of serious side effects, making autologous adipose SVF a promising tool in orthopedic care [18,19].

## Conclusions

To our knowledge, this is the first study to utilize this technique for non-traumatic talar AVN without it serving as an adjuvant to another surgical procedure. Given the existing data in the literature, one might hypothesize that combining core decompression with SVF could eventually result in better outcomes. However, in the present case, we opted not to proceed with core decompression due to the extensive nature of the lesion. Even though clinical improvement was not accompanied by radiological improvement, the symptomatic relief and positive impact on quality of life are undeniable. While this technique could become another tool in the conservative management of talar AVN, further research and documentation of similar cases will be essential for evaluating whether this technique truly offers a meaningful difference in the management of talar AVN, particularly as a conservative approach.
